# Chrysin loaded Nanostructured lipid carrier (NLC) drug delivery system: Harnessing neuroprotective effects in the management of Alzheimer’s Disease

**DOI:** 10.1002/alz.088755

**Published:** 2025-01-09

**Authors:** Nikhil B Khandale, Devendra S Birla, Bushra Bashir, Sachin Kumar Singh, Sukriti Vishwas, Monica Gulati, Kamal Dua

**Affiliations:** ^1^ School of Pharmaceutical Sciences, Lovely Professional University, Jalandhar, Punjab India; ^2^ Lovely Professional University, Jalandhar India; ^3^ Faculty of Health, Australian Research Centre in Complementary and Integrative Medicine, University of Technology Sydney, Ultimo, NSW, 2007, NSW Australia; ^4^ Discipline of Pharmacy, Graduate School of Health, University of Technology Sydney, Ultimo, NSW, 2007, NSW Australia

## Abstract

**Background:**

Alzheimer’s Disease (AD) poses a substantial global health burden, necessitating innovative therapeutic strategies. This study investigates the neuroprotective potential of a chrysin‐loaded Nanostructured Lipid Carrier (NLC) drug delivery system in AD management. Employing the high‐pressure homogenization method, chrysin‐loaded NLCs were meticulously formulated to optimize drug delivery efficiency. The research explores the multifaceted neuroprotective effects of chrysin, a naturally occurring flavonoid, within the context of AD pathology. The intricate mechanisms of action underlying the chrysin‐loaded NLCs are elucidated, shedding light on their potential to modulate key pathways implicated in AD progression. This study advances our understanding of tailored drug delivery systems, specifically highlighting the therapeutic promise of chrysin‐loaded NLCs for the nuanced management of Alzheimer’s Disease.

**Method:**

To overcome these challenges, a Nanostructured lipid carrier (NLC) for Chrysin was devised, employing quality by design (QbD) based Box‐Behnken design (BBD). NLC offers numerous advantages, encompassing increased drug loading, facile preparation, exceptional stability, as well as enhanced bioavailability and permeability across the blood‐brain barrier.

**Result:**

The optimized Chrysin‐loaded NLC formulation exhibited Particle Size, PDI, zeta potential, and drug loading as 66.45nm, 0.19, ‐22mV, and 98% respectively. The formulation was found to be stable after six months in accelerated stability studies. The formulation underwent various pharmacodynamic studies. Pharmacodynamic studies assessed cognitive and motor functions in rats, revealing significant improvements in cognition with both low and high doses respectively. Additionally, the biochemical investigations revealed that the Chrysin‐loaded NLCs effectively decreased the levels AChE enzyme, amyloid β, oxidative stress, and neuroinflammation.

**Conclusion:**

This study explores Chrysin‐loaded NLCs as a solution for challenges in Alzheimer’s Disease (AD) treatment. The optimized formulation overcomes Chrysin’s solubility issues, demonstrating improved bioavailability and permeability. Chrysin‐loaded NLCs hold promise as a neuroprotective for AD.

